# Different acquisition systems for heart rate variability analysis may lead to diverse outcomes

**DOI:** 10.1590/1414-431X2021e11720

**Published:** 2022-02-04

**Authors:** F.A. de Oliveira, R.A. Pereira, A.S. Silva, J.L. de Brito Alves, J.H. Costa-Silva, V.A. Braga, C.M. Balarini

**Affiliations:** 1 Departamento de Fisiologia e Patologia, Centro de Ciências da Saúde, Universidade Federal da Paraíba, João Pessoa, PB, Brasil; 2 Departamento de Educação Física, Centro de Ciências da Saúde, Universidade Federal da Paraíba, João Pessoa, PB, Brasil; 3 Departamento de Nutrição, Centro de Ciências da Saúde, Universidade Federal da Paraíba, João Pessoa, PB, Brasil; 4 Departamento de Educação Física e Ciências do Esporte, Universidade Federal de Pernambuco, Vitória de Santo Antão, PE, Brasil; 5 Centro de Biotecnologia, Universidade Federal da Paraíba, João Pessoa, PB, Brasil

**Keywords:** Heart rate variability, Spectral analyses, ECG

## Abstract

Heart rate variability (HRV) is a relevant physiological variable for the estimation of cardiac autonomic function. Although the gold standard for HRV registration is the electrocardiogram (ECG), several applications (APPs) have been increasingly developed. The evaluation carried out by these devices must be compatible with ECG standards. The aim of this study was to compare the data obtained simultaneously with ECG and APP with chest heart rate transmitters. Fifty-six healthy individuals (28 men and 28 women) were evaluated at rest through a short simultaneous HRV measurement with both devices. Data from both acquisition systems were analyzed separately using their own analysis software and exported and analyzed using a validated software. Signal recordings were compatible between the two acquisition systems (Pearson r=0.99; P<0.0001). Although a high correlation was found for the HRV variables obtained in the time domain (Spearman r=0.99; P<0.0001), the correlation decreased in the frequency domain (Pearson r=0.85; P<0.0001) when two software programs were used. Comparison of the averages of spectral analysis parameters also showed differences when HRV data were analyzed separately in each device for low-frequency (LF) and high-frequency (HF) bands. Although the portability of these mobile devices allows for optimal HRV evaluation, the direct analysis obtained from these devices must be carefully evaluated with respect to frequency domain parameters.

## Introduction

Heart rate variability (HRV) reflects oscillation in the intervals between consecutive heartbeats or RR intervals (distance between two successive R-waves) (1). HRV evaluation can provide extensive information regarding the autonomic modulation of cardiac function (2,3). Previous reports have demonstrated that HRV measurements and analysis are valuable tools for risk stratification and may predict the development of cardiovascular diseases (4-6) and metabolic disorders such as diabetes (7,8) and obesity (9,10). The applicability of HRV evaluation has also been demonstrated in neurology (11), anesthesia, and surgery (12). Furthermore, the use of HRV has expanded into the field of exercise physiology to assess the adaptability of the cardiovascular system to physical activity programs (3,13).

The advantage of being a noninvasive and easy-to-run test has encouraged studies of HRV, its application, and development (14-16). Since the establishment of guidelines for the use of HRV by the European Society of Cardiology and the North American Society of Electrophysiology (1), this technique has been increasingly applied, with a focus on the standardization of evaluation protocols and determination of reference values for the parameters obtained in the HRV analysis (17,18). Electrocardiogram (ECG) is the gold standard for recording RR intervals for subsequent HRV studies (18). However, owing to innovative technological advances, devices with greater portability, lower cost, easier operation, and increased accessibility have recently emerged as new tools for HRV recordings and analysis (19,20). The use of these devices, such as chest heart rate monitors (HRM), despite optimizing HRV evaluation, raises issues regarding the reliability and accuracy of the generated data. Although the reproducibility of HRV devices has been evaluated in different situations (21-23), whether their performance is equivalent to the gold standard remains controversial. In some validation studies, mobile devices synchronized with the transmitters are used only for signal acquisition (22,24,25). In these studies, HRV analysis is often performed by an external software. Therefore, the aim of this study was to further highlight the compatibility and reliability of these analyses by comparing HRV data obtained by two different recording methods (chest HRM and ECG) and analyzed using two different softwares. We evaluated the accuracy of signal recording using chest HRM and the reliability of data processing of a smartphone application (APP). Our findings confirmed that chest HRM can be used for recording purposes, but the analysis performed by the APP should be considered with caution, especially with regard to spectral analysis.

## Material and Methods

### Design and participants

In this cross-sectional study, each participant attended an experimental session to simultaneously record their heart electrical activity using ECG and chest HRM. The study was conducted in accordance with the ethical guidelines of the 1975 Declaration of Helsinki, as reflected in *a priori* approval by the institutional Ethics and Research Committee of the Federal University of Paraiba, Brazil (Protocol No. 2.303.755). Written informed consent was obtained from all volunteers before their participation. Fifty-nine healthy volunteers aged 18-50 years were recruited. Exclusion criteria were anatomic variations, use of artificial pacemakers, thoracic injuries, or other conditions that would interfere with the correct positioning of the elastic belt or ECG electrodes. The final evaluation included 56 participants (28 men and 28 women).

### HRV recordings

HRV measurements were performed at the Human Physiology Laboratory, Department of Physiology and Pathology, Health Sciences Center at the Federal University of Paraiba, Brazil. Initially, age, body mass, height, body mass index (BMI), and systolic and diastolic blood pressures using the auscultatory method were recorded. Participants also answered a short questionnaire regarding their level of physical activity. Recordings were performed during the day, at least 24 h after any physical exercise. All tests were performed with the participant at rest and in the supine position. A short-term recording was carried out for 10 min, and the participant was told to remain silent and breathe normally at tidal volume. The ECG 26T-LTS model (ADinstruments^®^, Australia) was used, and the recordings were made with the 5-electrode configuration using Labchart^®^ data acquisition software (ADinstruments^®^, version 8.1.6). The ECG was set to a sampling rate of 1 kHz and a range of 2 mV using a digital filter of 50 Hz (low pass). Simultaneously, a smartphone (iPhone, model 5S, IOS system version 11.3.1, Apple Inc., USA) was used to record the RR intervals on an APP (HRV Expert by Cardiomood^®^, version 1.6, Russia) through wireless transmission. An elastic belt of adjustable size was positioned comfortably around the participant's chest and a transmission device (Polar^®^ model H7, Polar Electro, Finland; 5 kHz transmission system coded with Owncode^®^, Polar Electro) was attached on the front at the level of the xiphoid process of the sternum. The RR intervals obtained using Cardiomood^®^ were exported to the manufacturer's web platform and ECG data were exported to Labchart. Both sets of data were also analyzed directly by each systems' programs.

**Figure 1:**
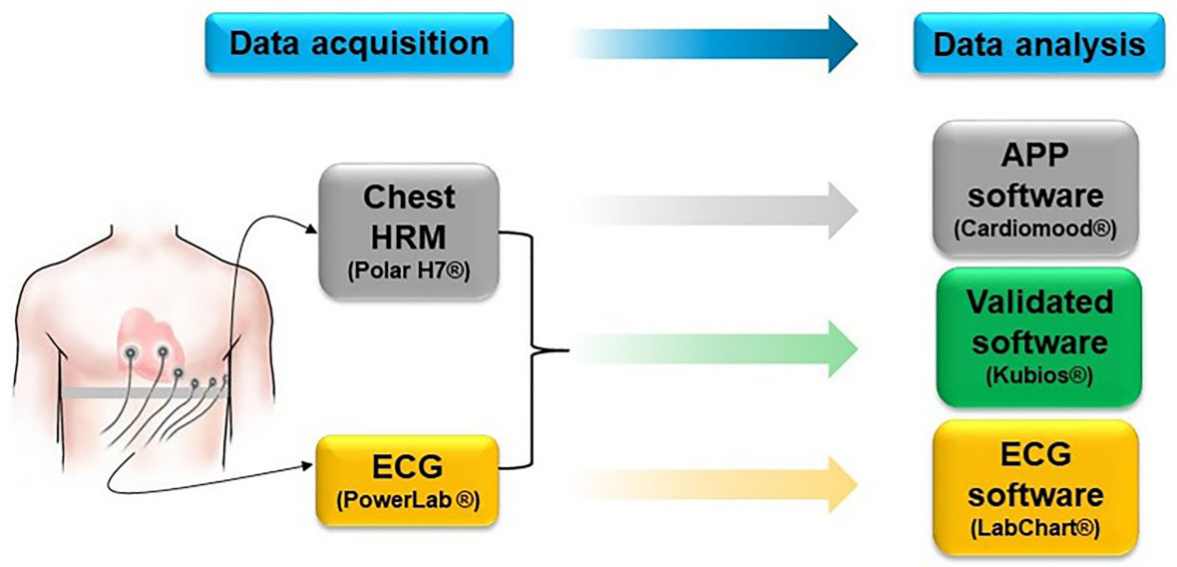
Illustration of the research strategy. Blue flowchart: Division between data acquisition and data processing/analysis process; Grey flowchart: Data acquisition by the attached chest device (heart rate monitors - HRM) with analysis in the mobile phone application (APP); Yellow flowchart: Data acquisition by the coupled electrocardiogram (ECG) with analysis in the ECG software; Green flowchart: Data acquisition by coupled ECG and chest device with analysis with a validated common external/alternative software.

Recordings were made for 10 min. The first 5 min were considered the stabilization period and discarded, and the last 5 min of each recording were used for comparison purposes. Both devices were handled together, and the recordings were performed simultaneously. Data obtained by both systems were separately analyzed on each device's analysis software and exported and analyzed with Kubios HRV standard software version 3.0.2 (Biomedical Signal and Medical Imaging Analysis group, Department of Applied Physics, University of Kuopio, Finland) (Figure 1). In this software, frequency domain analysis was conducted using a fast Fourier transformation (FFT) and threshold medium, and the interpolation of the series was performed by cubic spline. The frequency values were set to 3 hz.

The HRV evaluation included the following parameters in the time domain: standard deviation between the duration of RR intervals (SDNN, ms) and root mean square differences of successive RR intervals (RMSSD, ms). We also evaluated the following variables in the frequency domain: low-frequency band (LF, from 0.04 to 0.15 Hz) and high-frequency band (HF, from 0.15 to 0.40 Hz). The power of each spectral component was calculated using normalized units (nu). Normalization was performed by dividing the power of each band by the total power, from which the very low frequency (VLF, 0.0033-0.04 Hz) band value was subtracted and the result was then multiplied by 100 (26).

### Statistical analysis

Data were subjected to the Shapiro-Wilk normality test and then evaluated using Pearson or Spearman correlation tests as appropriate. For mean comparison, the unpaired *t*-test was used. Geometric means (back-transformed means of the log-transformed data) were calculated for the time domain variables. The agreement between methods for every derived HRV parameter was assessed using Bland-Altman technique (27). Further, paired *t*-test was used to test significant differences between parameters derived from the two methods. Linear regression analyses were applied to assess biases of proportionality adopting the average values of each software's measures as independent variables and the differences between both measures as dependent variables. Data are reported as means±SD for the profile variables of volunteers and as means and 95% confidence intervals for HRV parameters. The significance level was set at P<0.05.

## Results

**Figure 2:**
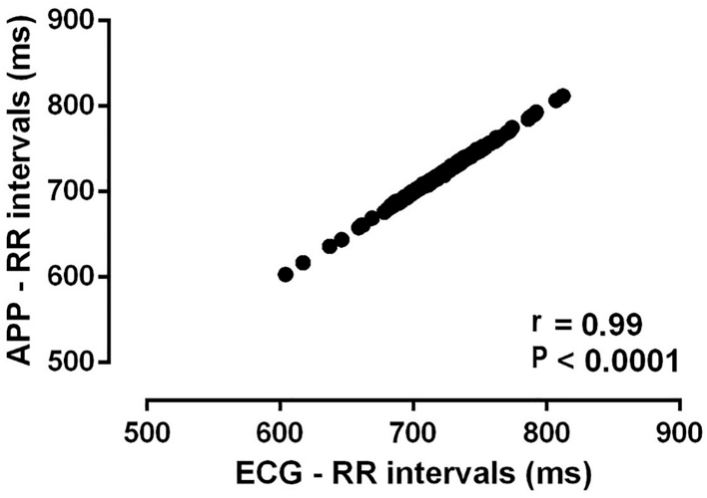
Correlation between RR intervals obtained simultaneously during a 5-min period by electrocardiogram (ECG) and by mobile phone application (APP). The data are shown as averages of each interval [number of XY pairs=404]. Pearson's correlation was performed with 95% confidence interval (0.99 to 0.99).

The general characteristics of the 56 participants are presented in Table 1. The profile of the participants revealed that the study population consisted of young, eutrophic, and normotensive adults. Additionally, most individuals reported doing physical activity (33.8% regularly and 35.4% irregularly), while 30.8% did not perform physical exercises. Notably, data from the two devices were similar. Correlation analysis of the RR intervals showed that the records obtained with the two systems had a high level of correlation (r=0.99, P<0.0001, Figure 2).

**Table 1:** Characteristics of the healthy individuals (28 men and 28 women) evaluated for heart rate variability simultaneously using smartphone application and electrocardiogram.

Variables	Mean±SD (n=56)
Age (years)	23.5±9.1
Height (cm)	169±9
Body mass (kg)	65.6±11.5
Body mass index (kg/m^2^)	22.7±3.3
Heart rate (bpm)	71.2±7.6
Systolic blood pressure (mmHg)	114.5±10.7
Diastolic blood pressure (mmHg)	72.8±9.6

The SDNN values ranged from 23.2 to 95.5 milliseconds and RMSSD values from 14.2 to 116.5 milliseconds. Our findings demonstrated diverse HRV within the population studied. Despite the apparent HRV heterogeneity, the correlation between the simultaneous recordings by the devices was very strong (r=0.99 and P<0.0001). This pattern was maintained when analyses were performed separately in each software, as well as when using a common external software.

**Figure 3:**
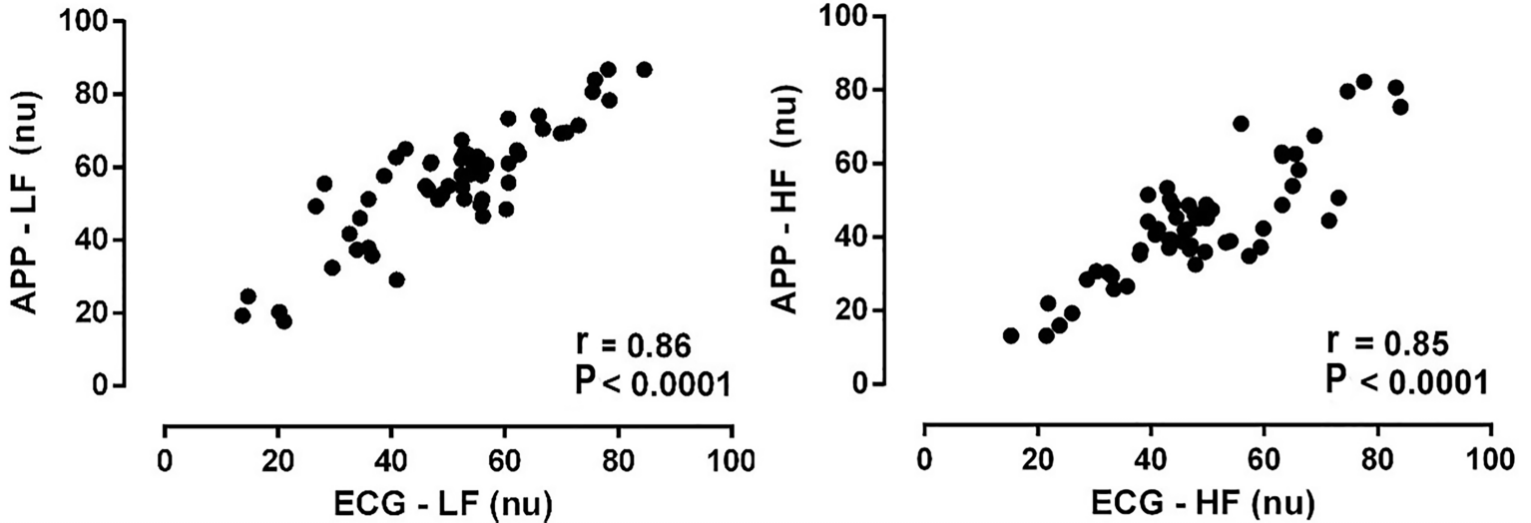
Correlation between the frequency domain variables [low and high frequency (LF and HF)] separately calculated by each software [application (APP) and electrocardiogram (ECG)]. Data are reported as means of each registry [n=56]. Pearson's correlation was performed and the 95% confidence intervals were LF=(0.78 to 0.91) and HF=(0.76 to 0.91). nu: normalized units.

**Figure 4:**
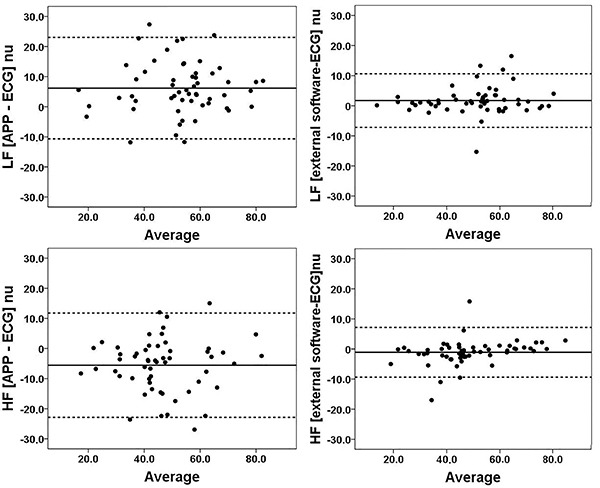
Bland-Altman agreement analysis between electrocardiogram (ECG), smartphone application (APP), and external software measurements of the frequency domain bands (low and high frequency - LF and HF). Solid lines indicate average differences and dotted lines refer to 95% limits of agreement (±1.96*SD). nu: normalized units.

The variables in the frequency domain were also studied (Figure 3). As observed for the parameters in the time domain, the average HF and LF bands also maintained a high level of correlation when analyzed using the same software (r=0.99 and P<0.0001). However, when the evaluation was performed separately in each software, considerable dispersion was observed and the correlation levels for both LF and HF decreased (r=0.86 and 0.85, respectively). Inter-device reliability for LF and HF bands was evaluated using Bland-Altman plots (Figure 4) and showed differences in the analysis methods for obtaining these parameters (Table 2). The linear regression results for proportionality bias in absolute differences and average values (LF and HF nu) between APP, ECG, and external analyzes can be viewed in Table 3. The mean values of each variable were compared in addition to the correlation analyses (Table 4). Consistently, a significant difference was found between the parameters in the frequency domain (LF and HF) when the analysis was performed using different softwares. There was no difference between means in the time domain.

**Table 2:** Parameters analyzed by the Bland-Altman technique.

	Bias and [SD of bias]	95% LoA (lower and upper)	P value
LF (APP - ECG)	6.17 [8.60]	-10.68 and 23.04	<0.001
LF (external software - ECG)	1.73 [4.54]	-7.16 and 10.62	0.006
HF (APP - ECG)	-5.54 [8.84]	-22.88 and 11.79	<0.001
HF (external software - ECG)	-1.09 [4.22]	-9.37 and 7.17	0.057

Data are reported in normalized units (nu). P<0.05 paired *t*-test was used to test significant differences between parameters derived from the two methods (APP *vs* ECG or external software *vs* ECG). LF: low-frequency band; HF: high-frequency band; APP: smartphone application software; ECG: electrocardiogram software; 95% LoA: 95% limits of agreement.

**Table 3:** Linear regression for proportionality bias in absolute differences and average values (LF and HF nu) between APP, ECG, and external analyses.

	Adjusted R^2^	β	P value
LF (APP - ECG)	-0.017	0.037	0.785
LF (external software - ECG)	-0.001	0.132	0.330
HF (APP - ECG)	0.018	0.025	0.852
HF (external software - ECG)	0.077	0.306	0.022

Linear regression analyses were applied to assess biases of proportionality adopting the average values of each software's measures as independent variables and the differences between both measures as dependent variables (P<0.05). LF: low-frequency; HF: high-frequency; nu: normalized units; APP: smartphone application software; ECG: electrocardiogram software.

**Table 4:** Comparison of the heart rate variability parameters obtained separately with a smartphone application (chest HRM) and electrocardiogram (ECG) and analyzed with a common or different analysis software.

Parameters	Same software			Different software		
	ECG	Chest HRM	P value	ECG	Chest HRM	P value
SDNN (ms)	51.9 [46.9-56.8]	51.9 [47.4-56.8]	0.97	51.9 [46.9-56.8]	51.9 [47.4-56.8]	0.93
RMSSD (ms)	43.4 [38.5-49.4]	43.4 [38.5-48.8]	0.97	43.4 [39.6-48.8]	43.4 [38.5-48.8]	0.96
LF (nu)	51.7 [47.7-55.8]	52.7 [48.6-56.8]	0.99	50.0 [46.0-54.0]	56.2 [52.1-60.3]	0.03*
HF (nu)	48.1 [43.9-52.3]	47.1 [43.0-51.3]	0.74	49.2 [45.3-53.0]	43.6 [39.7-47.5]	0.04*

Data are reported as mean (95%CI) for n=56. SDNN: standard deviation between the duration of RR intervals; RMSSD: root mean square differences of successive RR intervals; nu: normalized units. For SDNN and RMSSD, the means were back-transformed (geometric means). *P*<*0.05, unpaired *t*-test.

## Discussion

With the advancement of HRV analysis, different devices have been developed and tested, with portable transmission devices being found to be reliable (28-30). The HRV analyses of the various applications under development must be tested and proven to be equally reliable. However, the reliability of the records obtained using mobile devices and their potential applicability in clinical evaluation and scientific research remains controversial. In the present study, we compared HRV data obtained using chest HRM with data obtained with the gold standard (ECG). Herein, the signal quality obtained with the chest HRM was found to be reliable, but the data processing by the APP software was not reliable and should be used with caution.

Although the profile of the participants examined in this study revealed that the sample cohort was composed of young, eutrophic, and normotensive adults, our primary aim was not to evaluate the HRV of these participants in relation to their own health status, age, or gender, but instead to compare HRV analyses obtained using two different recording methods, regardless of possible individual differences between participants. Based on the wide spectrum of the obtained HRV recordings, the comparison can be valid for any group of participants with high or low variability. Furthermore, the mean values obtained in this study are in agreement with the reference values of other studies (18,31,32).

The RR intervals and HRV parameters in the time and frequency domains were compared and correlated. First, we evaluated the quality of the signal generated by the chest HRM. This was possible because of a simple strategy: the recordings of RR intervals obtained with each device (chest HRM and ECG) were exported and processed by the same external software, previously validated for HRV analysis (29). The original tachogram obtained using the two different systems showed a high similarity between the tracings over time. Consequently, the correlation between the obtained RR intervals was high. This finding suggested that the recording and signal transmission of RR intervals were equivalent in both acquisition systems. Because the tachogram is a graphical representation of instantaneous RR intervals obtained over time, it can be assumed that both devices detect the same variations between RR intervals. A study using the same chest HRM suggested that the mobile approach could modify the signal during pre-processing (33). Based on the present findings, we believe that the quality of the signal is sufficiently reliable, once a consistent match was found between the data obtained with the two devices when analyzed using the same software (both in time and frequency domains). Of note, the compatibility between data from the two devices was consistent despite the diverse range of individual values for each parameter in the correlation line (indicating various levels of HRV among participants). The records obtained using chest HRM or ECG were equivalent for participants with higher or lower HRV parameters.

The second phase of the study involved data processing using the APP's or the ECG's own analysis software. A decrease in the correlation values of the parameters in the frequency domain (LF and HF) was found between the devices used. A recent comparative study (24) demonstrated excellent compatibility between the smartphone APP and the ECG. In addition, agreement was observed for all parameters in the time domain, frequency domain, and nonlinear indices. However, in that study, the devices were used only as signal recorders and the obtained data were compared a single analysis software. In the current study, we demonstrated that the data obtained by the two methods are only consistent for the time domain parameters (we did not evaluate non-linear parameters). The frequency domain parameters (HF and LF) obtained by the ECG and by the APP, without interference from other programs, showed significant variations. Thus, portability of the APP method was attested only for time-domain analyses and not for frequency-domain analyses.

As expected, a high level of correlation was found between RR intervals and consequently between the parameters in the time domain obtained with the two methods. This compatibility occurred regardless of the software used for analyses (common/single software or obtained by each device's software) because, as mentioned, they are calculated from a set of identical RR intervals. However, the weak correlation between the frequency domain parameters analyzed within each device indicated that data processing for spectral analysis may differ between the devices. Considering the Bland-Altman plot, it can be inferred that there is a difference between the analysis methods (non-concordant models). However, the analyses performed with the external software had a greater similarity. This observation is reinforced by the proximity of the mean plot line to zero on the difference axis, but also by the widening of the limits of agreement when the methods are compared. The Kubios^®^ software (external software used to analyze the data from the ECG and the mobile device) uses Welch's periodogram and provides results analyzed by the autoregressive method. For comparison, the results obtained by Welch's periodogram (FFT spectrum) were used. Welch's periodogram is not suitable for 24-h spectral analysis because it uses an FFT width of 256 s, which does not reflect the HRV of the full period (34). However, in the current study, a short registration period was used, which does not result in any loss in the analysis capacity of this periodogram. The company Labchart^®^ (ECG software) certifies that the software uses the Lomb periodogram for spectral analysis. The Lomb-Scargle periodogram is a spectral estimation technique appropriate for time series with irregular samples using the original data (35), and this signal treatment provides a spectral estimate of HRV with less noise. However, we could not obtain information about the data processing used in the Cardiomood^®^ software. Sometimes, softwares on mobile devices are real “black boxes” and do not provide details about the analysis/parameters used for data processing. Thus, the post-processing signal management may be a cause of disparities, as equivalent HRV signals result in different values for frequency domain parameters (33). This may contribute to the tendency to assign advantages of time-domain parameters over frequency-domain parameters in HRV interpretation (36).

The technological advancement for accessing biomedical signals seems to be a very promising field, and the portability offered by various devices has shown considerable potential. It would be an important development for clinical practice, especially in health systems focused on primary care, if the arsenal of diagnostic tools adapted to mobile applications (thus making assessment more accessible and portable) would have a high and reliable level of accuracy to enable better quality of preventive follow-up at home.

Regarding HRV, our data suggested that the chest HRM and the smartphone APP can acquire the signal correctly compared to the gold standard [ECG]. However, the APP apparently did not reproduce the HRV analysis identically to the ECG in the frequency domain, although it is fully compatible with the variables in the time domain. Considering the conditions under which the measurements were performed and the manner in which the data were processed, chest HRM can be recommended for data recording purposes. The evaluation of variables in the time domain using the APP is compatible with the results obtained using the gold standard and can be viewed as reliable. However, for a more complete analysis of HRV parameters, including the variables in the frequency domain, data processing of RR intervals obtained using chest HRM should be performed by a recognized external HRV analysis software.
